# Influence of prior illness on exertional heat stroke presentation and outcome

**DOI:** 10.1371/journal.pone.0221329

**Published:** 2019-08-20

**Authors:** Michelle A. King, Matthew D. Ward, Thomas A. Mayer, Mark L. Plamper, Clifford M. Madsen, Samuel N. Cheuvront, Robert W. Kenefick, Lisa R. Leon

**Affiliations:** 1 Thermal and Mountain Medicine Division, United States Army Research Institute of Environmental Medicine, Natick, MA, United States of America; 2 John H. Bradley Branch Health Clinic, Marine Corps Base, Quantico, VA, United States of America; University of Roehampton, UNITED KINGDOM

## Abstract

**Introduction:**

Precipitating factors that contribute to the severity of exertional heat stroke (EHS) are unclear. The purpose of this study was to determine the effect of prior illness (PI) on EHS severity.

**Methods:**

We performed a retrospective clinical record review of 179 documented cases of EHS at the Marine Corps Base in Quantico, Virginia.

**Results:**

Approximately 30% of EHS cases had a medically documented PI. Anthropometrics (height, weight, body mass index) and commonly associated risk factors for EHS (age, number of days in training, wet bulb globe temperature, sleep patterns) did not differ between PI and no illness (NI) groups. PI patients presented with higher maximal rectal core temperatures (40.6 ± 1.0°C vs. 40.3 ± 1.2°C; P = 0.0419), and elevated pulse rates (118.1 ± 16.7 bpm vs. 110.5 ± 24.2 bpm; P = 0.0397). At the point of care, biomarker values were similar between PI and NI groups, with the exception of a trend toward elevated monocytes in those with PI (7.9 ± 2.9% vs 6.7± 2.7%; P = 0.0521). Rate and duration of cooling were similar between PI and NI patients.

**Conclusion:**

This study indicates that PI has a minimal effect on the patient presentation, severity and treatment outcome of EHS. The results of this study have important implications for military, civilian, and occupational populations who are at risk for EHS.

## Introduction

Exertional heat stroke (EHS) is a serious condition that affects military and civilian populations alike, and if left untreated, results in death. EHS is commonly defined by central nervous system (CNS) dysfunction accompanied by a body core temperature (Tc) of ≥40°C, although the specific Tc associated with this definition varies, and diagnoses have been made at Tcs as low as 38.4°C [1–3]. The etiology of EHS is still largely unknown, and there are a variety of proposed risk factors that contribute to both the incidence and severity of the condition [4–7]. The most commonly studied risk factors pertain to environmental conditions and the physical fitness of the individual [8, 9]. Interestingly, individuals often suffer from EHS without any known risk factors [10–12]. To understand this phenomena, focus has shifted toward lesser known risk factors for EHS, such as prior illness (PI) or infection [13–15]. Evidence suggests that recent or concurrent illness may not only increase the likelihood of experiencing EHS, but also increase the severity of the injury [16].

The hypothesis that recent or concurrent illness affects the likelihood and severity of EHS is primarily derived from retrospective studies in humans and supported by animal research [7, 14, 15, 17]. When exposed to a simulated immune response, rodents undergoing acute heat stress attained higher maximal rectal core temperatures (Tc max) and exhibited diminished capacity to tolerate heat stress [16]. Decreased rates of survival and amplified biomarker profiles indicated that severity of injury was exacerbated by the simulated infection [15, 16]. By extension, this suggests that PI may also prolong cooling times if, in response to infection, fever has elevated the thermoregulatory set point. However, this concept has not yet been investigated.

Multiple ideas have been put forth as to how PI, infection, or an immune disturbance may contribute to EHS susceptibility [13]. One theory states that when immunity is suppressed for an extended time period, i.e. during prolonged intense physical training, an infection or immune disturbance may perturb the body’s ability to manage heat stress. This perturbation may exaggerate the pro-inflammatory and pyrogenic cytokine response which increases Tc further [13, 18]. Other theories propose that PI elevates baseline Tc which becomes additive in EHS and that the direct effect of higher Tc is responsible for the multi-organ dysfunction witnessed in EHS [10]. High tissue temperatures lead to systemic loss of plasma membrane integrity, which contribute to the initiation and magnitude of organ injury that occurs in EHS [19–22]. Rectal Tc is used as an estimate of internal tissue temperature in human EHS victims, and while specific tissue temperatures vary [23, 24], it provides an estimate for the probable heat-induced cytotoxicity.

To determine how PI affects patient presentation, blood biomarker profiles, and response to cooling at the point of care following EHS, we performed a clinical record review of documented EHS cases at the Marine Corps Base in Quantico, Virginia. In this rare clinical data set, physiological variables were recorded from the point of care until the cessation of the standardized treatment protocol in a homogenous population. The standardization and careful documentation of patient data allowed for an in depth analysis of the EHS episode and treatment outcomes that is not available in civilian mass participation settings or arenas. Based on experimental animal studies and anecdotal human evidence, we hypothesized that individuals who experienced PI prior to or concurrent with the EHS episode would present with a higher Tc max, protracted cooling times, and exhibit adverse biomarker profiles in comparison to individuals without a documented illness (NI).

## Methods

A retrospective clinical record review of EHS was carried out at the Officer Candidates School Marine Corps Base in Quantico, Virginia. This study included 179 eligible cases of heat casualty reports treated at the Bradley Branch Naval Health Clinic during the years of 2012–2015. Data were collected from the NAVMED 6500/1, the standard heat or cold injury reporting form for Navy Bureau of Medicine. These paper records were copied, redacted of personally identifiable information, and entered into a database for later statistical analyses. The appropriate institutional review boards approved this study and deemed this research exempt from obtaining written informed consent (United States Army Research Institute Environmental Medicine & Unites States Army Medical Research and Materiel Command).

### Description of treatment protocol

A retrospective description of the treatment protocol is described here as documented by the John H. Bradley Branch Health Clinic physicians. EHS cases identified on the training field by altered mental status and a Tc of ≥40°C were promptly (typically less than 5 minutes) transported to the Naval Health Clinic (directly adjacent to the training field) for treatment. There, an emergent cooling protocol was initiated if deemed appropriate by medical staff [25, 26]. This treatment protocol consisted of standardized cooling where individuals were continuously doused with ice water and the lower extremities actively massaged with ice bags. The axilla, neck, and groin were also cooled by placement of ice bags in these areas. The cooling treatment was accompanied by convective overhead fan cooling. A blood draw was taken immediately upon arrival for assessment of blood biomarkers. The cooling treatment ceased at the discretion of the provider (typically 102°F/ 38.9°C). Cooling duration was determined as the amount of time from the initiation of the emergent cooling protocol until the time of cooling cessation. Rate of cooling was determined by subtracting the final Tc from the initial Tc and dividing this by the total amount of cooling time. The standard of care was the same for all patients. EHS patients were identified in the field by medical staff and were included for analysis if evidence was provided that the patient received the cooling treatment protocol.

Rectal Tc (measured via rectal thermistor; (DataThermII, Geschwenda, Germany) accuracy: ± 0.1°C: 34.0–42.0° and ± 0.2°C: for other ranges) was obtained at the point of injury in the field and immediately upon presentation to the clinic (i.e. point of care or treatment). Tc max was considered the highest value of these two recordings. In accordance with standard operating procedures, the cooling treatment was rapidly initiated upon arrival to the clinic. Once intravenous access was established, the primary blood sample was taken. Patient vitals (blood pressure, heart rate, saturation of oxygen, respiration rate) and rectal Tc were recorded immediately upon presentation and serially throughout the course of treatment. A detailed patient history of variables surrounding the injury was recorded after emergent treatment was completed, and the patient was stabilized. These variables included: age, height, weight, body mass index (BMI), duration of sleep the previous night (hours), description of last meal, and amount of water (quarts) within the last 12 hours. The wet bulb globe temperature index (WBGT) was obtained from historical weather records on the base.

### Categorization of prior illness

Individuals were categorized as PI if the provider indicated recent illness or infection anywhere within patient history. Although this categorization included all illnesses with varying onsets prior to EHS, this broad inclusion criteria was intended to detect long lasting effects of viral or bacterial infections that lacked clinically overt symptoms. Further, some individuals were simply marked as “recent illness” by the medical provider without documentation of the specific number of days that the illness occurred prior to EHS. In this way, our criteria included anything the provider documented as “recent illness” although we utilize the term “PI” to describe the range in onset of illness.

### Measured variables (blood analytes)

iSTAT (handheld blood analyzer) (Abbott laboratories, Chicago, IL) was used to determine electrolyte concentration, (sodium (Na^+^), potassium (K^+^), chloride (Cl^-^), ionized calcium (iCa), anion gap (AnGap)) kidney function, (blood urea nitrogen (BUN), creatinine) blood gases (total carbon dioxide(TCO_2_)), glucose, hematocrit, and hemoglobin concentrations in the blood. The complete blood count (CBC) panel included white blood cells (WBC), white blood cells corrected (WBC Corrected), red blood cells (RBC), hemoglobin, hematocrit, mean corpuscular volume (MCV), mean corpuscular hemoglobin (MCH), mean corpuscular hemoglobin concentration (MCHC), red blood cell distribution width (RDW CV), platelets, mean platelet volume (MPV), neutrophils, lymphocytes, monocytes, eosinophils, basophils, and absolute values of the these blood cells, with nucleated red blood cells per 100 white blood cells (NRBC/100WBC). Plasma enzymes and markers of cell damage were measured as a part of the metabolic panel (albumin, alkaline phosphatase (ALP), alanine aminotransferase (ALT), bilirubin, glomerular filtration rate (GFR) (black and non-black), aspartate aminotransferase (AST)). Other laboratory markers of cell damage were ordered independently (creatine kinase (CK), lactate dehydrogenase (LDH), and urate).

### Statistics and data analysis

Statistical analyses were performed using SAS JMP (Cary, NC) and Graphpad Prism (La Jolla, CA). Univariate analysis was utilized in this dataset because qualifications for multi-variate analysis were not met. Welch’s Test for unequal variance was used to compare differences between PI and NI groups. Results are presented as mean ± standard deviation with the 95% confidence interval (CI). A linear regression was used to examine the relationship between Tc max, clinical biomarkers, number of recovery days, and the duration or rate of cooling. A *P-value* of ≤0.05 was considered statistically significant. A *P-value* of <0.08 was considered a trend. *Data completeness*: The specific type and number of clinical labs obtained for each patient were at the discretion of the provider and varied between patients. To account for this, unequal variance testing was utilized.

## Results

To test our hypothesis, 179 records (of the 208 collected) were included for analyses. Twenty-nine patients were excluded from analyses because there was no evidence that the patient received the emergent cooling treatment. This data set was comprised of 36 female and 143 male patients. Patient information including physical characteristics, vital sign measurements, clinical laboratory values, and environmental conditions associated with the episode are presented in Tables 1 and 2.

**Table 1:** EHS Patient Characteristics, Vital Signs, and Risk Factors Taken at the Point of Care.

	TOTAL	NI	PI	P Value
**Physical Characteristics**	n = 179	n = 126	n = 53	
Age (years)	22.8 ± 3.1	22.8 ± 3.1	22.8 ± 3.0	0.9814
BMI (kg/m^2^)	24.6 ± 2.5	24.5 ± 2.4	24.9 ± 2.6	0.3122
Height (cm)	174 ± 9.5	174 ± 9.6	174 ± 9.2	0.7368
Weight (kg)	74.9 ± 11.3	74.4 ± 11.8	75.9 ± 10.1	0.4013
**Environmental Risk Factors**				
Days in Training (#)	29.8 ± 32.0	31.1 ± 37.0	27.3 ± 18.8	0.4362
Meals Prior (hrs)	7.4 ± 5.4	6.6 ± 5.2	9.2 ± 5.5	0.0251*
Prior Sleep (hrs)	4.6 ± 1.8	4.6 ± 1.9	4.7 ± 1.6	0.8224
Water Consumption (qts)	5.3 ± 3.6	5.0 ± 3.4	6.2 ± 4.0	0.0654
WBGT (°C)	22.4 ± 5.3	22.5 ± 4.7	22.2 ± 6.5	0.7450
**Patient Vital Signs**				
Diastolic BP (mmHg)	63.1 ± 11.6	64.0 ± 11.5	60.9 ± 11.5	0.1002
MAP (mmHg)	80.2 ± 17.8	80.1 ± 18.6	80.5 ± 16.0	0.8913
Pulse (bpm)	112.8 ± 22.4	110.5 ± 24.2	118.1 ± 16.7	0.0184*
Respiration (bpm)	22.3 ± 10.1	22.4 ± 11.2	22.0 ± 7.2	0.7934
Systolic BP (mmHg)	122.2 ± 15.9	121.2 ± 14.9	124.4 ± 18.0	0.2667
Tc Max (°C)	40.4 ± 1.2	40.3 ± 1.2	40.6 ± 1.0	0.0419*
**Cooling Treatment**				
Cooling Duration (min)	12.3 ± 6.7	12.2 ± 6.6	12.5 ± 7.1	0.7918
Cooling Rate (°C/min)	0.20 ± 0.3	0.20 ± 0.3	0.17 ± 0.1	0.2968

Values are mean ± SD. Welch’s Test for Unequal Variance.

*P≤0.05

**Table 2:** Clinical Laboratory Values Taken at the Point of Care.

Analyte	TOTAL	NI	PI	P Value	Resting Ref Range
Na (mmol/L)	139.3 ± 3.5	140.0 ± 4.0^#^	139.6 ± 2.5	0.8751	(138–146)
K (mmol/L)	4.0 ± 0.5	4.0 ± 0.6^#^	4.0 ± 0.4	0.8529	(3.5–4.9)
Cl (mmol/L)	104.4 ± 3.8	104.2 ± 4.0	105 ± 3.4	0.2667	(98–109)
iCa (mg/dL)	4.5 ± 0.3	4.5 ± 0.3	4.5 ± 0.4^#^	0.9994	(4.5–5.3)
TCO2 (mmol/L)	16.0 ± 5.6	16.4 ± 5.6	14.8 ± 5.5	0.1493	(24–29)
Glucose (mg/dL)	151.6 ± 64.0	145.7 ± 63.5	165.7 ± 63.8	0.1292	(70–105)
BUN (mg/dL)	16.0 ± 5.5	16.4 ± 6.1	15.1 ± 3.9	0.1815	(8–26)
Creatinine (mg/dL)	1.5 ± 0.4	1.5 ± 0.4^#^	1.5 ± 0.4^#^	>0.9999	(0.6–1.3)
AnGap (mmol/L)	24.3 ± 5.6	23.9 ± 5.3	25.1 ± 6.1	0.3408	(10–20)
Hematocrit (%)	40.4 ± 4.0	41.0 ± 4.0	39.4 ± 3.7	0.1652	(37–52)^ [27]
Hemoglobin (g/dL)	13.7 ± 1.3	14.0 ± 1.4	13.4 ± 1.3	0.1593	(12–18)^ [27]

Values are mean ± SD. Welch’s Test for Unequal Variance. Reference ranges were obtained from the iSTAT handheld blood analyzer.

^Denotes reference ranges were unavailable from iSTAT and were taken from the indicated reference.

^#^Denotes median and IQR are reported for nonparametric data.

### Incidence of prior illness

PI was reported in 29.6% of patients, with the most common infection identified as upper respiratory tract infection (URTI). The average onset of infection was approximately nine days before the EHS episode, but this varied from the day of EHS (18% of patients) to a singular case of bronchitis diagnosed 56 days before the episode. Specific types of PI or infections listed in the patient history are included in Table 3.

**Table 3:** Number and type of recent illness documented.

Recent Illness Diagnosis Included:	Count:	Average PI Onset (Days Prior to EHS)	Range of PI Onset (Days Prior to EHS)
Upper Respiratory Infection	13	6.1	0–23
Cellulitis	8	5.5	2–10
“Cold” Non Specified	7	2.7	0–6
Bronchitis	5	16.7	0–56
Sinusitis	5	7.8	0–14
Pneumonia	3	0	0
Rhabdomyolysis	3	34.5	30–39
Blisters	2	21	21
Diarrhea	2	1	0–2
Flu	2	27.7	9–39
Cold Symptoms	5	8.2	0–25
Febrile illness	1	17	17
Folliculitis	1	5	5
Gastroenteritis	1	5	5
Gout	1	n/a	n/a
Hematuria	1	1	1
Ingrown toe nail	1	32	32
Pink eye	1	3	3
Positive PPD	1	7	7

Fourteen individuals had more than one PI listed, while six did not have a number of days prior to EHS listed for the illness. “0” days prior to EHS indicates that this illness was present on the same day as EHS.

### EHS presentation and severity

Patient characteristics, vital signs, and blood biomarkers were compared between those diagnosed as PI and NI. No differences in baseline characteristics (age, weight, height, and BMI) were present between groups. Risk factors such as WBGT, acclimatization (number of days in training), and sleep patterns were also similar (Table 1). PI patients tended to display disturbances in immune function by a trend toward increased monocytes (7.9 ± 2.9% vs 6.7± 2.7%; P = 0.0521; 95% CI -2.48 to 0.01) and neutrophil depletion (57.3 ± 15.9% vs. 63.6 ± 15.2%; P = 0.0736; 95% CI -0.62 to 13.21) (S1 Table). PI patients reported changes in eating patterns, including an increase in the amount of time since the last meal was consumed (9.2 ± 5.2 hrs. vs 6.6 ± 5.2 hrs.; P = 0.0251; 95% CI 0.34 to 4.89) (Table 1). Among the remaining patient characteristics, vital signs, and blood biomarker patterns, pulse was the only variable that distinguished between PI and NI groups, with a higher pulse recorded in PI at presentation, likely a result of the increased Tc in this group (118.1 ± 16.7 bpm vs. 110.5 ± 24.2 bpm; P = 0.0397; 95% CI 1.31 to 13.96) (Table 1).

### Activity at the time of collapse

One hundred and fifty (84%) of the 179 records documented the activity at the time of EHS collapse. The most commonly reported activity (33%) was the “Endurance Course” (≈ 5km with six obstacles to traverse), followed by hikes ranging in distance from 6km-19km (4–12 miles) (18%), and fartlek runs (period of fast running intermixed with periods of slower running) (13%).

### Clothing at the time of collapse

Although only 90 (~50%) of the 179 records analyzed indicated the type of clothing worn at the time of collapse, the most commonly listed clothing was the “full combat uniform” (38%), followed by “uniform” (31%), “physical training gear” (24%), and “fartlek gear” (7%). This is not surprising as the full combat uniform provides the most coverage and additional weight load; whereas physical training gear and fartlek gear (likely shorts and a T-shirt) provide the least amount of coverage.

### Response to cooling treatment

**Fig 1:**
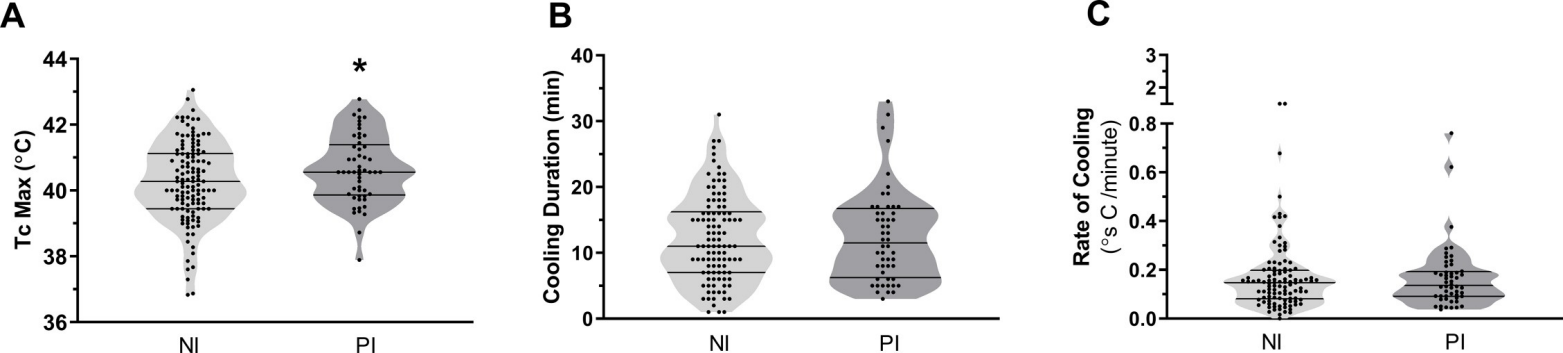
Violin plots representing response to cooling. **(**A) Tc max (rectal maximal core temperature in degrees Celsius), (B) cooling duration (minutes cooled), and C. Rate of cooling (degrees Celsius per minute). Tc max is significantly higher in PI whereas rate of cooling and duration are similar between groups. Welch’s Test for unequal variance, *P≤0.*05*.

**Fig 2:**
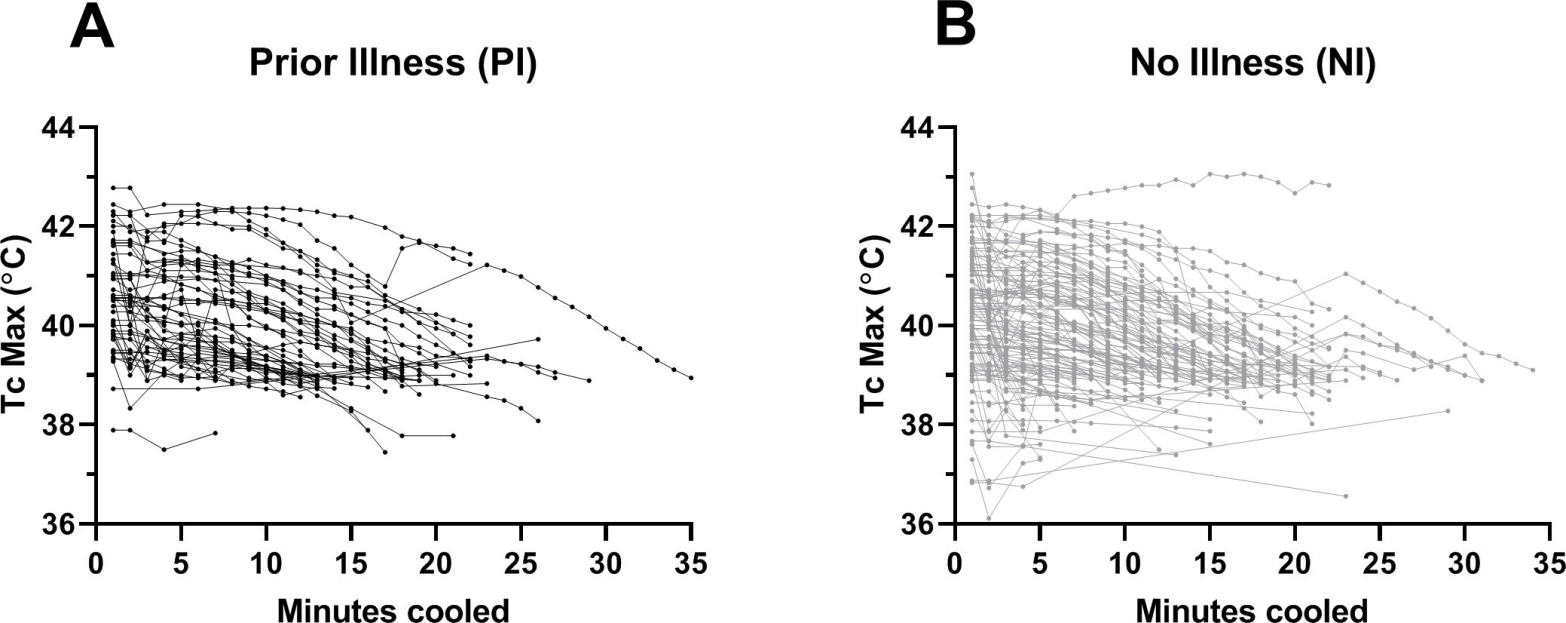
Individual cooling patterns for those with or without PI. Rectal core temperature (Tc) was taken to assess rate and duration of cooling in patients with prior illness (PI) (A) and those without (NI) (B). The individual Tc at select time points is represented for each individual.

PI patients presented with a higher Tc max (P = 0.0419; 95% CI 0.01 to 0.72), however PI had no effect on the duration (P = 0.7918; 95% CI -2.03 to 2.65) or rate of cooling (P = 0.2968; 95% CI -0.1 to 0.03) (Fig 1, Table 1). Tc max was positively correlated with the duration of cooling (R^2^ = 0.4354; P<0.0001; 95% CI 2.09 to 3.0) but not with the rate of cooling (R^2^ = 0.0002, P = 0.8515; 95% CI -0.02 to 0.02) perhaps due to the large variability in Tc and cooling durations. The individual cooling pattern (cooling curve) is presented in Fig 2. Cooling curves display each individual’s rate of decline in Tc from the initiation of cooling until cessation of the treatment protocol. There was no association between rate of cooling and biomarkers at the time of presentation or outcomes such as return to duty.

### Return to duty

Following EHS, patients were prescribed a certain number of days “sick in quarters” or recovery days. This information was recorded for 58% of this population. Of those recorded, 91% were prescribed one rest day, while 7% were prescribed two rest days and the remaining 2% were prescribed three rest days. The number of recovery days before patients returned to duty did not differ with PI (P = 0.2898; 95% CI -0.09 to 0.29).

## Discussion

This study demonstrated that although Tc max was increased in patients with PI, this did not alter the response to cooling or the severity of EHS as indicated by patient vital signs, the blood biomarker profile taken at the point of care, or recovery days. Until now, the influence of PI on the severity of EHS has not been studied in a controlled manner in a human population. To our knowledge, this is the first data set to document sequential recordings of Tc and patient vitals over the course of a standardized EHS cooling treatment protocol. Further, this robust dataset provided detailed patient histories, which allowed us to make conclusions about the influence of risk factors on the clinical presentation and response to treatment. This study is largely generalizable to the population most at risk for EHS [28, 29] and adds an essential clinical perspective to literature that has largely involved rodent data or anecdotal evidence.

In this population of officer candidates, 29.6% reported some type of PI, which included any condition considered an immune disturbance, ranging from upper respiratory tract infections (URTI) to blisters (Table 3). Despite blood biomarker values falling largely within the normal reference range, immune cell populations (e.g. monocytes and neutrophils) that are commonly altered in response to infection were discernible between PI and NI (S1 Table). Besides distinctions in immune cell populations, appetite suppression and elevated heart rates also supported the medical provider classification of prior illness. Data shows that the prevalence of PI (URTI, flu, diarrhea) in a military population is approximately 16–18% [2, 30]. The larger percentage in this study may be due to the extended period of intense training that officer candidates are exposed to, which has been shown to suppress the immune system and increase the incidence of PI [31–33] or the inclusion criteria. High rates of PI are also common in athletic teams undergoing intense training and living in close quarters where communicable infection is easily spread [34]. Despite the high prevalence of documented PI in the officer candidates, this was not related to the severity of EHS as indicated by vital signs (respiration and blood pressure) or the blood biomarker profile.

Subclinical fever is a symptom of recent illness which may predispose to or exacerbate severity of EHS [13, 18, 35, 36] and potentially alter the response to treatment. The Tc max of the PI group was approximately 0.3°C above the NI group, which proved to be of little consequence in EHS severity (Tables 1 and 2). This small, but significant, elevation in Tc did not affect the response to the cooling treatment protocol. We observed that the pattern, rate, and duration of cooling were not altered by PI (Fig 1A). It appears that the ability of the body to respond to the cooling treatment protocol was very similar between PI and NI. Cooling duration in the current study averaged approximately 12 minutes at a rate of 0.22°C/min (0.39°F/min) for the entire population (Table 1). It has been suggested that rates of cooling should be 0.1–0.2°C/min (0.18–0.36°F/min) to ensure the best patient outcome [37]. Although other cooling methods have been utilized that achieve similar rates of cooling [38], this study supports the effectiveness of the field cooling treatment protocol and the positive patient outcomes. The cooling technique described here has not yet been widely referenced in the literature. However, the positive patient outcomes speak to the validity of this cooling treatment, and it should be recommended in tactical situations when appropriate. This study further highlights that prompt initiation of cooling is the most critical aspect of ensuring positive treatment outcomes regardless of the exact cooling protocol.

Evidence from animal models demonstrate that PI reduces heat tolerance and amplifies the severity of injury by increases in inflammatory cytokines, circulating injury biomarkers (ALT, AST, Creatinine, BUN), and death [15, 16]. Evidence for differences in circulating biomarkers of organ injury was not supported in the current study, where clinical biomarker profiles taken at the point of care were similar between PI and NI groups. Given the lack of a blood draw prior to EHS, it is unknown how biomarker profiles were influenced by PI prior to EHS. Previous research has demonstrated that biochemical changes which discriminate the severity of EHS may not be visible until after 24 hours following the EHS event [39]. In the present study, follow up blood draws were not available for analysis. Of those documented, the majority (91%) of patients returned to duty within one day of the EHS event, implying that biomarker profiles returned to normal. This return to duty is accelerated compared to the US Army recommendations [40]. However, this study highlights that rapid identification and treatment of EHS may prevent organ damage and shorten recovery. Future studies should obtain information from serial blood draws over the course of treatment to better inform return to duty decisions. Therefore, our data elucidate that any acute effect of PI on blood biomarkers may be of little clinical importance when attempting to predict severity of injury or course of treatment outcomes at the point of care.

### Considerations

The influence of PI on the blood biomarker profile and Tc max may have been confounded by the exercise intensity and duration at the time of collapse. Blood biomarkers taken at the point of care for EHS are often indistinguishable from those expressed following an exhaustive bought of exercise [41]. Although exercise intensity was not measured, the training events at the time of injury were similar types of endurance activities across patients. Another challenge to data such as these is ensuring that inclusion criteria correctly identify EHS cases and exclude other heat illness cases. Because this is a retrospective dataset, the initial diagnosis by medical providers was retained. Some of the recorded temperatures observed in this study are considerably lower than those typically considered EHS, although it is unknown if the diagnoses for these individuals changed retrospectively based on spontaneous cooling or the biomarker profile. More importantly, this highlights that reliance on Tc for diagnosis of EHS is suboptimal and more sensitive and specific criteria should be met in order to diagnose EHS.

## Conclusion

This is the first study to demonstrate the impact of PI on patient presentation, the blood biomarker profile, and response to cooling at the point of care following EHS. We describe a 30% PI rate among training Marine officer candidates and for the first time, demonstrated that PI patients present with a higher Tc max. In contrast to animal studies and popular anecdote in humans, our primary finding demonstrated that PI did not have a significant role in influencing the severity of EHS (blood biomarkers and thermoregulation) beyond a higher Tc max. While PI appears to be a common occurrence in individuals that experience EHS, the effect of PI on the severity of this condition appears minimal.

## Practical implications

From a clinical perspective, PI has little influence on the normal course of treatment that follows EHS. However, the increase in Tc that accompanies PI may impose a greater physiological strain on organ tissues, which may have consequential effects if patients are left untreated.

## Supporting information

S1 Table Other clinical lab values taken at point of care. (PDF)

S1 File Original database provided for availability of data. (XLSX)
