# Supplementary material for: Influence of prior illness on exertional heat stroke presentation and outcome
Source: PLoS One. 2019 Aug 20;14(8):e0221329. doi: 10.1371/journal.pone.0221329 (PMC6701802; doi:10.1371/journal.pone.0221329)
Supplement: S1 Table — (PDF) [file pone.0221329.s001.pdf]

S1 Table. Other clinical lab values taken at point of care

Analyte	TOTAL	NI	PI	P value	Resting Ref Range
Albumin (g/dL)	4.6 ± 0.5	4.5 ± 0.6	4.7 ± 0.6	0.4129	(3.5-5.5)
ALK P (U/L)	73 ± 16.5	84.7 ± 18.5	75.1 ± 17.9	0.0555	(36-126)
ALT (U/L)	43.6 ± 14.1	62.9 ± 50.3	44.8 ± 16.2	0.0518	(7-56)
AST (U/L)	92.9 ± 82.0	95.6 ± 68.1	71.6 ± 20.5	0.0554	(17-49)
Bilirubin (mg/dL)	0.9 ± 0.4	0.9 ± 0.5	0.8 ± 0.3	0.3848	(0.2-1.3)
Urea Nitrogen (mg/dL)	13.2 ± 3.3	14.4 ± 3.3	13.5 ± 3.9	0.3862	(9-21)
Protein (g/dL)	7.1 ± 0.6	7.2 ± 0.7	7.2 ± 0.7	0.9539	(6.3-8.2)
GFR NB (mL/min/1.73m^2)	68.9 ± 17.8	70.0 ± 15.0	67.1 ± 21.9	0.5910	(≥90)^
GFR B (mL/min/1.73m^2)	82.4 ± 20.9	85.2 ± 15.8	78.4 ± 26.6	0.3268	(≥90)^
LDH (U/L)	794.8 ± 183.4	770.7 ± 233.0	734 ± 127.6	0.4472	(313-618)
Urate (g/dL)	7.6 ± 1.5	7.3 ± 1.6	7.0 ± 0.9	0.2857	(3.3-8.4)
CK (U/L)	2197 ± 5255	1438 ± 3092.1	951.8 ± 859.1	0.2407	(55-170)
**Complete Blood Count**					
WBC (x10^3/mcL)	10.7 ± 3.7	11.0 ± 3.5	10.4 ± 4.0	0.5003	(3.6-10.6)
WBC Corrected	10.5 ± 3.5	10.7 ± 3.3	10.1 ± 3.7	0.4334	(4-11)
RBC (x10^3/mcL)	4.6 ± 0.5	4.6 ± 0.5	4.5 ± 0.4	0.2900	(4.21-5.92)
Hemoglobin (g/dL)	13.8 ± 1.3	13.7 ± 1.3	13.8 ± 1.2	0.7446	(12.8-17.7)
Hematocrit (%)	41.5 ± 4.6	41.5 ± 3.6	41.3 ± 6.1	0.8095	(37.5-50.9)
MCV (fL)	91.5 ± 5.6	90.6 ± 5.6	93.1 ± 5.4	0.0500*	(79.5-96.8)
MCH (pg)	30.1 ± 2.3	29.8 ± 2.5	30.5 ± 2.0	0.1466	(26.2-33.1)
MCHC (%)	33.0 ± 1.1	33.1 ± 1.1	32.8 ± 1.2	0.3136	(32.6-35.0)
RDW (%)	13.6 ± 1.0	13.6 ± 1.2	13.5 ± 0.6	0.4355	(12-16.2)
Platelets (x10^3/mcL)	280.1 ± 63.4	280.6 ± 64.9	279.1 ± 61.2	0.9128	(162-427)
MPV (fL)	9.4 ± 1.0	9.5 ± 1.0	9.2 ± 1.0	0.1197	(7.0-10.9)
Neutrophils (%)	61.5 ± 15.6	63.6 ± 15.2	57.3 ± 15.9	0.0736	(40.7-76.4)
Lymphocytes (%)	29.2 ± 14.2	27.4 ± 13.9	32.6 ± 14.5	0.1073	(15.9-47.8)
Monocytes (%)	7.1 ± 2.8	6.7 ± 2.7	7.9 ± 2.9	0.0521	(4.5-11.8)
Eosinophils (%)	1.1 ± 0.9	1.0 ± 1.0	1.4 ± 1.3	0.1009	(0-4.0)
Basophils (%)	0.7 ± 0.5	0.7 ± 0.5	0.8 ± 0.4	0.4245	(0-4.0)
ABS Neutrophils (x10^3/mcL)	6.7 ± 3.3	7.1 ± 3.3	5.9 ± 3.1	0.0836	(1.8-7.5)
ABS Lymphocytes (x10^3/mcL)	3.4 ± 5.3	2.8 ± 1.6	3.1 ± 2.0	0.4240	(1.0-3.1)
ABS Monocytes (x10^3/mcL)	0.8 ± 0.5	0.8 ± 0.4	0.9 ± 0.6	0.2726	(0.2-0.8)
ABS Eosinophils (x10^3/mcL)	0.1 ± 0.1	0.1 ± 0.1	0.1 ± 0.1	0.2544	(00.0-0.5)
ABS Basophils (x10^3/mcL)	0.1 ± 0.1	0.1 ± 0.1	0.1 ± 0.1	0.9031	(0.0-0.4)
Nucleated RBC/100 WBC	0.1 ± 0.1	0.1 ± 0.1	0.1 ± 0.1	0.4206	1

Values are mean ± SD. Welch's Test for Unequal Variance. **P≤0.05*

^Denotes reference ranges were unavailable and were taken from reference [27].
